# Association between faecal load of *lawsonia intracellularis* and pathological findings of proliferative enteropathy in pigs with diarrhoea

**DOI:** 10.1186/1746-6148-8-198

**Published:** 2012-10-23

**Authors:** Ken Steen Pedersen, Marie Ståhl, Roberto Maurício Carvalho Guedes, Øystein Angen, Jens Peter Nielsen, Tim K Jensen

**Affiliations:** 1 HERD – Centre for Herd oriented Education, Research and Development, Department of Large Animal Sciences, University of Copenhagen, Groennegaardsvej 2, Frederiksberg C 1870, Denmark; 2 National Veterinary Institute, Technical University of Denmark, Bülowsvej 27, Copenhagen V 1790, Denmark; 3 Department of Veterinary Clinics and Surgery, Veterinary School, Universidade Federal de Minas Gerais, Av. Antônio Carlos 6627, Belo Horizonte, MG, 31.270-901, Brazil

**Keywords:** Diarrhoea, Immunohistochemistry, *Lawsonia intracellularis*, Quantitative real time PCR, Pig

## Abstract

**Background:**

The study was designed to investigate correlation between histological findings of *Lawsonia intracellularis* in porcine cases of diarrhoea and the quantitative detection of *Lawsonia intracellularis* in faeces. A total of 156 pigs (10 to 70 days post weaning) with diarrhoea were randomly selected from 20 herds: The pigs were subjected to necropsy, histopathology, immunohistochemistry and faecal quantification of *Lawsonia intracellularis* by real time PCR.

**Results:**

The median *Lawsonia intracellularis* excretion was significantly higher in pigs with gross lesions of proliferative enteropathy (median excretion: 5.92 log_10_ bacteria/g faeces) compared to pigs without gross lesions of proliferative enteropathy (median excretion: <3.3 log_10_ bacteria/g faeces) (*P*<0.001). Spearman’s correlation coefficient between the measureable PE lesions and *L. intracellularis* excretion was 0.50 (*P*<0.001). A significantly increasing trend in *Lawsonia intracellularis* excretion level for increasing proliferative enteropathy histopathology and immunohistochemistry scores was demonstrated (*P*<0.001; *P*<0.001). Spearman’s correlation coefficient between the histopathology scores and *L. intracellularis* excretion was 0.67 (*P*<0.001). Spearman’s correlation coefficient between the IHC scores and *L. intracellularis* excretion was 0.77 (*P*<0.001).

**Conclusions:**

The histological and quantitative PCR detection of *Lawsonia intracellularis* were correlated in pigs with diarrhoea. Overall the results suggest that clinically important levels for *Lawsonia intracellularis* excretion in faeces may be established. Such clinical threshold levels may be used in practice to confirm a diagnosis of *Lawsonia intracellularis* associated diarrhoea.

## Background

The antibiotic consumption in the agricultural industry has increasing concern in relation to development of antimicrobial resistance in both animals and humans [1]. In Denmark approximately 50% of the antibiotics in pigs is used for treatment of diarrhoea [2]. Optimizing antibiotic treatments or reducing disease occurrence necessitates a correct diagnosis. A number of intestinal pathogens have been associated with diarrhoea in growing pigs, including *Escherichia coli, Brachyspira pilosicoli, Brachyspira hyodysenteriae*, *Salmonella* spp. and *Lawsonia intracellularis*. *L. intracellularis* is the cause of proliferative enteropathy (PE) and considered one of the most important intestinal pathogens [3]. Clinical signs associated with the different intestinal pathogens are similar and microbiological investigations are necessary to confirm the specific microbiological cause in cases of diarrhoea. For *L. intracellularis* obtaining a correct microbiological diagnosis is complicated. Subclinical infection is common [4], *L. intracellularis* can be demonstrated by PCR in animals vaccinated by an avirulent live vaccine [5] and the bacterial load in faeces could previously not be assessed in routine diagnostic work as the bacterium could only be cultivated and maintained in cell cultures [6]. Development of quantitative PCR (qPCR) tests for quantification of *L. intracellularis* in faeces [5,7-10] has now made it possible to determine the number of *L. intracellularis* bacteria in faeces on a routine basis. This may be useful for confirmation of *L. intracellularis* as the microbiological cause in porcine diarrhoea. Correlation between disease severity and excretion load for a specific pathogen may be expected. However, such correlation cannot always be demonstrated [11].

In this paper a quantitative real time PCR was used to investigate correlation between histological findings of *L. intracellularis* in porcine cases of diarrhoea and the quantity of *L. intracellularis* detected by qPCR in faeces

## Results

A total of 20 outbreaks were examined (one outbreak in 20 different herds) and 160 pigs (mean days post weaning = 33) were euthanized during the herd visits. Four pigs were excluded as less than 1.0 gram of faeces had been obtained, providing a total of 156 pigs with diarrhoea for the statistical analysis.

*L. intracellularis* was detected by qPCR in 25.6% of the pigs. Among the qPCR positive pigs 30.0% were below the dynamic range (4.3 log10 bacteria/g faeces) of the qPCR, 70.0% were in the dynamic range and no pigs were above the dynamic range (8.3 log10 bacteria/g faeces). The median excretion for all positive pigs was 5.44 log_10_ bacteria/g faeces.

### Gross pathology

**Figure 1:**
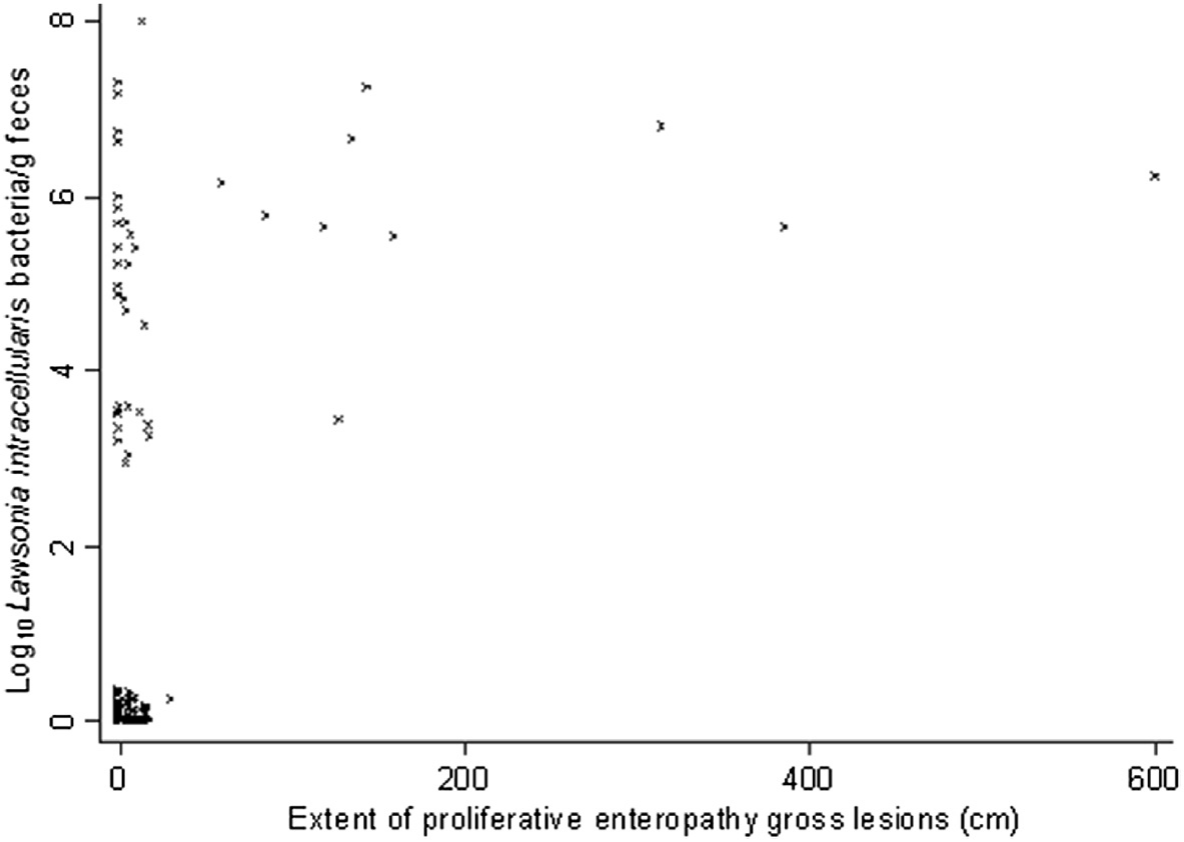
**Association between proliferative gross lesions and *Lawsonia intracellularis *excretion. **Association between measureable proliferative intestinal gross lesions and *Lawsonia intracellularis* excretion levels in faeces determined by qPCR from pigs with diarrhoea (n=156).

Gross lesions of PE were demonstrated in 8.3% of the pigs. The mean gross lesions in pigs with measureable lesions (*n*=11) were 193 cm in extent (range: 17 to 600). Among the pigs without gross lesions, 20.3% were qPCR positives (median excretion: 5.03 log_10_ bacteria/g faeces). Among the pigs with gross lesions, 84.6% were qPCR positives (median excretion: 6.01 log_10_ bacteria/g faeces). Spearman’s correlation coefficient between the measureable PE lesions and *L. intracellularis* excretion was 0.50 (*P*<0.001). Association between the measureable PE lesions and *L. intracellularis* excretion in qPCR positive pigs are displayed in Figure 1. The median *L. intracellularis* excretion was significantly higher in pigs with gross lesions (median excretion: 5.92 log_10_ bacteria/g faeces) compared to pigs without gross lesions (median excretion: below limit of detection) (*P*<0.001).

### Histopathology

Histological lesions of PE were demonstrated in 14.1% of the pigs. Among the pigs without histological lesions, 14.9% were qPCR positives (median excretion: 4.41 log_10_ bacteria/g faeces). Among the pigs with histological lesions, 90.9% were qPCR positives (median excretion = 6.0 log_10_ bacteria/g faeces.

**Figure 2:**
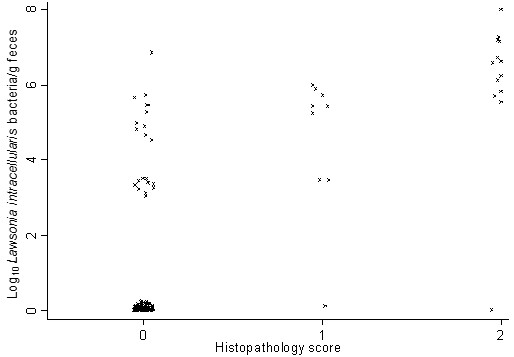
**Association between histopathology and *Lawsonia intracellularis *excretion. **Association between histopathology score and *Lawsonia intracellularis* excretion levels in faeces determined by qPCR from pigs with diarrhoea (n=156).

The Kruskal-Wallis equality of populations rank tests demonstrated an overall significantly difference between the median excretion levels for the histopathology scores (*P*<0.001). The test for trend demonstrated a significantly increasing trend in excretion level for increasing histopathology scores (*P*<0.001). Spearman’s correlation coefficient between the histopathology scores and *L. intracellularis* excretion was 0.67 (*P*<0.001). Association between the histopathology score and *L. intracellularis* excretion is displayed in Figure 2.

### Immunohistochemistry

**Figure 3:**
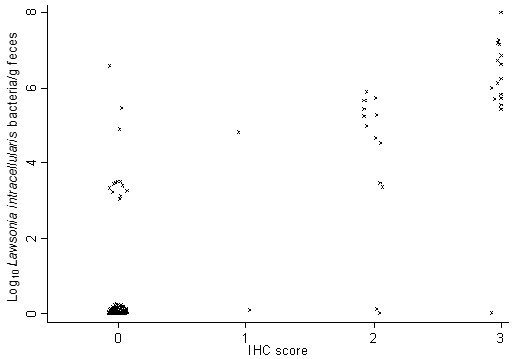
**Association between immunohistochemistry (IHC) and *Lawsonia intracellularis *excretion. **Association between immunohistochemistry (IHC) score and *Lawsonia intracellularis* excretion levels in faeces determined by qPCR from pigs with diarrhoea (n=156).

*L. intracellularis* was detected by IHC in 19.9% of the pigs. Among the IHC negative pigs, 10.4% were qPCR positives (median excretion: below dynamic range). Among the IHC positive pigs, 87.1% were qPCR positives (median excretion = 5.83 log_10_ bacteria/g faeces). The Kruskal-Wallis equality of populations rank tests demonstrated an overall significantly difference between the median excretion levels for the IHC scores (*P*<0.001). The test for trend demonstrated a significantly increasing trend in excretion level for increasing IHC scores (*P*<0.001). Spearman’s correlation coefficient between the IHC scores and *L. intracellularis* excretion was 0.77 (*P*<0.001). Association between the IHC score and *L. intracellularis* excretion is displayed in Figure 3.

## Discussion

Routine q-PCR quantification of a number of virus infections is now well established in medicine and has recently been reviewed [12-14]. Application of qPCR for quantification of bacterial infections is less well established in routine diagnostics, especially in veterinary medicine. However, correlation between disease severity and qPCR quantification of bacterial infections in clinical samples from animals and humans has been reported for *Borrelia burgdorferi*[15], *Mycoplasma genitalium*[16], *Brucella* spp. [17], *Helicobacter pylori*[18], *Mycobacterium leprae*[19,20], *Mycoplasma gallisepticum*[21], *Streptococcus pneumoniae*[22], *Brucella melitensis*[23], *Haemophilus influenzae*[24] and *Legionella penumophila*[25]. Further, clinical cut-off levels for bacterial load has been established for *Streptococcus pneumoniae*[22,26], *Haemophilus influenzae*[24], *Mycobacterium tuberculosis*[27], *Gardnella vaginalis* and *Atopobium vaginae*[28].

In the current study a positive correlation between disease severity in terms of pathological findings and quantitative detection of *L. intracellularis* in faeces was demonstrated in pigs with diarrhoea. These results are in accordance with a previous report of correlation between *L. intracellularis* bacteria load in mucosal scrapings and the severity of intestinal lesions [29]. In qPCR positive pigs the median *L. intracellularis* excretion level was higher in pigs with PE gross lesions compared to pigs without gross lesions. This suggests that quantification by qPCR might be applied for examination of presence or absence of gross lesions of PE. The extent of PE gross lesions and *L. intracellularis* excretion levels were apparently not correlated. However, this association should be further investigated because of the low number of pigs with gross lesions in the current study.

In contrast, increasing histopathology/IHC scores were correlated to increasing *L. intracellularis* excretion levels. One interesting aspect of the observed observations is that demonstration of *L. intracellularis* in faeces is not evident of ileitis but in case of ileitis high excretion levels of *L. intracellularis* can be expected. The applied study design did not take progression of *L. intracellularis* infection into consideration. The obtained results are only relevant for the association between gross pathology, histopathology, IHC and quantification of *L. intracellularis* by qPCR at the time of faecal sampling. Correlation over time and progression of *L. intracellularis* excretion during an infection should be performed in a longitudinal study design. Factors that potential could have influenced the results are the intermitted shedding of *L. intracellularis* previously described [30] and non-homogeneous distribution of *L. intracellularis* in faeces and/or intestinal tissue. Quantification of *L. intracellularis* in faeces has been reported to have an acceptable within day repeatability [31] suggesting that non-homogeneous distribution of *L. intracellularis* in faeces is not a major bias in the current study. Some of the qPCR negative animals were probably false negatives because of intermitted shedding caused by a *L. intracellularis* excretion level close to the qPCR’s limit of detection as previously described [31]. This aspect has potentially introduced misclassification bias in the study. However, it is most likely only low excreting pigs that were classified as false negatives introducing only minor bias in the ranking of animals, the statistical analysis and the results. Inclusion of multiple standardized intestinal segments and inclusion of any gross lesions in the histological examinations have probably decreased any effect of a potential non-homogeneous distribution of *L. intracellularis* in the intestinal tissue.

Other factors including intestinal infections could potentially influence the reported associations. The reported lesions (IHC, proliferative lesions) are very specific for *L. intracellularis*. Therefore it seems very unlikely that other factors would have a confounding effect between these lesions and the faecal *L. intracellularis* excretion. However it is possible that interactions exists providing different absolute quantitative associations between *L. intracellularis* and the lesions depending on simultaneous infections or other factors. The number of observations in the current dataset did not allow for investigation of this aspect, which should be explored before clinical relevant threshold levels can be used in practice.

## Conclusions

The study has demonstrated that histological and quantitative PCR detection of *L. intracellularis* is correlated in pigs with diarrhoea. Overall the results suggest that clinically important levels for *L. intracellularis* excretion in faeces may be established. Such clinical threshold levels may be used in practice to confirm a diagnosis of *L. intracellularis* associated diarrhoea, for monitoring of treatment effects or for prognostic purposes.

## Methods

### Collection of materials from porcine diarrhoea

No prior information concerning faecal excretion levels of *L. intracellularis* in pigs with different levels of histological findings of *L. intracellularis* was available for a formal sample size calculation. General sample size and power considerations for estimation of correlation and differences between groups for continuous outcome variables were performed using Stata IC version 11. Data previously collected for investigation of outbreaks of treatment-requiring diarrhoea in pigs 10–70 days post weaning was considered to contain a sufficient number of pigs for the current study. In brief, farmers from 32 selected herds were requested to notify the corresponding author at initiation of an outbreak of diarrhoea in pigs between 10 and 70 days post weaning. All herds were visited the day following notification and the farmer was not allowed to medicate any pigs before the pigs had been examined.

To avoid any post antibiotic effect the outbreaks were not included if the pigs had received antibiotic medication in feed or water with-in the last 7 days. In each outbreak 8 pigs with diarrhoea was selected by random sampling for necropsy and collection of faecal samples. The pigs were euthanized in the herd, the abdomen was incised and intestinal specimens for histopathological examinations were collected. At the end of each herd visit the euthanized pigs and faecal samples were transported to the National Veterinary Institute, Technical University of Denmark. Pigs and faecal samples were stored at 4^o^ C. Necropsy and processing of faecal samples were performed the following day.

All parts of the study were performed in consistency to the Danish welfare legislation.

### Gross pathology

The pigs were subjected to necropsy and evaluation of internal organs. Gross lesions of PE was graded as 0 = no lesions, 1 = thickened intestinal wall and/or mucosa hyperplasia, 2 = necrotic enteritis. Furthermore, the extension of PE gross lesions was measured.

### Histology

Intestinal samples were obtained within 5 minutes of euthanasia and immediately fixated in 10% neutral buffered formalin. The intestinal tissue samples were obtained from ileum (approximately 5 cm from the ileo-caecal junction), one random site from jejunum and from the mid-spiral region of colon.

At necropsy further tissue samples for histology were obtained from jejunum (approximately 1.5 m from the ileo-caecal junction) and from any intestinal gross lesions. All samples were embedded in paraffin wax and sectioned in 3 μm sections.

All segments were stained by hematoxylin and eosin (H&E) for histopathology. Histological lesions of PE were graded on a scale from 0 to 4: Grade 0: no significant lesions; grade 1: up to 25% hyperplastic enterocytes, focal or multifocally, and reduction of the number of goblet cells; grade 2: 25 to 50% hyperplastic enterocytes, multifocally, and reduction of the number of goblet cells; grade 3: 50 to 75% hyperplastic enterocytes, multifocally, and reduction of the number of goblet cells; grade 4: More than 75% hyperplastic enterocytes, multifocally or diffusely, and reduction of the number of goblet cells.

All segments were stained by immunohistochemistry for detection of *L. intracellularis* as previously described [32]. Results of the IHC examination were graded on a scale 0–8: Grade 0: no labelled antigen; grade 1: Up to 25% of intestinal mucosa with labelled antigen in lamina propria only; grade 2: 25 to 50% of intestinal mucosa with labelled antigen in lamina propria only; grade 3: 50 to 75% of intestinal mucosa with labelled antigen in lamina propria only; grade 4: more than 75% of intestinal mucosa with labelled antigen in lamina propria only; grade 5: Up to 25% of intestinal mucosa with labelled antigen, enterocytes and lamina propria; grade 6: 25 to 50% of intestinal mucosa with labelled antigen, enterocytes and lamina propria; grade 7: 50 to 75% of intestinal mucosa with labelled antigen, enterocytes and lamina propria; grade 8: more than 75% of intestinal mucosa with labelled antigen, enterocytes and lamina propria.

### Faecal dry matter

All faecal samples were subjected to faecal dry matter determination to confirm the diagnosis of diarrhoea in the individual pigs. The individual faecal samples were mixed with a spoon and faecal dry matter content was determined by drying to constant weight using a microwave oven as previously described [33]. Only pigs with faecal dry matter content below 0.18 were considered diarrhoeic in the statistical analysis as previously described [33].

### qPCR

A suspension of 10% faeces in phosphate buffered saline (PBS) was prepared from each faecal sample. The individual faecal samples were mixed with a plastic spoon and 0.1 gram of faeces was suspended in 0.9 gram of PBS. The faeces suspension was stored at -80°C until further processing. Total DNA was extracted from the 10% faecal suspensions by using QIAsymphony extraction robot and QIAsymphony Virus/Bacteria Mini Kit (QIAGEN, GmbH, Germany). The protocol was Pathogen Complex 200, the processed sample volume was 200μL and elution was done in 110μL. Prior to DNA extraction the 10% faecal suspensions were pre-treated by bead beating in Tissuelyser (20 sek., 15 Hz, at room temperature, QIAGEN) with stainless steel beads (5 mm, QIAGEN). The suspensions were centrifuged for 90 sek. at 10 000 rpm (MiniSpin plus, Eppendorf) and the supernatant was transferred to the QIAsymphony robot. One negative extraction sample of other bacterial cells and one positive extraction sample of *L. intracellularis* were included in each experiment. All qPCR experiments were run in duplicates as previously described [9]. The limit of detection was 3.3 Log_10_ bacteria/g faeces and the dynamic range was 4.3 - 8.3 Log_10_ bacteria/g faeces.

### Statistical analysis

Classification of individual pigs in relation to histological lesions of PE and IHC was performed before the statistical analysis. The highest grade from any intestinal segment in a pig defined the grade for that pig. To obtain more observations in each category, both histopathology and IHC grades were reclassified in the statistical analysis. In relation to histopathology, pigs without histopathological lesions of proliferative enteropathy were classified as score zero. Pigs with histopathology grade one or two were classified as score one and pigs with histopathology grad three or four were classified as score two. In relation to IHC, pigs observed to be negative by IHC were classified as score zero. Pigs with labeled antigen in lamina propria only (IHC grade one, two or three) were classified as score one. Pigs with IHC grad four or five were classified as score two and pigs with IHC grad six or seven were classified as score three. Overall differences in the median excretion level between pigs with different scores of gross pathology, histopathology and IHC classifications was assessed by the Kruskal-Wallis equality of populations rank test. The overall trend in excretion levels for the different scores was assessed as previously described [34] and Spearman’s correlation coefficients including associated *P*-values were calculated.

## Competing interest

The authors declare that they have no competing interests.

## Authors' contributions

All authors conceived and designed the study. KSP performed all clinical herd investigations. RG and TKJ performed all pathological and histopathological examinations. MS and ØA performed all PCR experiments. KSP conducted the statistical analyses. All authors participated in drafting the manuscript. All authors have read and approved the final manuscript.
